# Evaluation of Cycle Threshold (Ct) Values for Detecting High-Risk HPV in Self-Collected Vaginal Samples as a Triage Method to Colposcopy

**DOI:** 10.3390/diagnostics15243205

**Published:** 2025-12-15

**Authors:** Kimon Chatzistamatiou, Menelaos Zafrakas, Glykeria Gkoliou, Electra Sofou, Konstantinos Pasentsis, Georgios Karakatsoulis, Theodoros Agorastos, Kostas Stamatopoulos

**Affiliations:** 1 Department of Obstetrics and Gynecology, Papageorgiou General Hospital, Aristotle University of Thessaloniki, 56403 Thessaloniki, Greece; 2 School of Health Science, International Hellenic University, 57001 Thessaloniki, Greece; 3 Institute of Applied Biosciences, Centre for Research & Technology Hellas, 57001 Thessaloniki, Greece; glykgkol@gmail.com (G.G.); electrasofou@gmail.com (E.S.); kpasents@certh.gr (K.P.); g.karakatsoulis@certh.gr (G.K.); kostas.stamatopoulos@certh.gr (K.S.); 4 Department of Obstetrics and Gynecology, Aristotle University of Thessaloniki, 54642 Thessaloniki, Greece; agorast@auth.gr

**Keywords:** HPV self-sampling, high-risk HPV, triage, colposcopy, Ct value, cycle threshold

## Abstract

**Background/Objectives:** The aim of this study was to evaluate the cycle threshold (Ct) values of self-collected vaginal samples as a triage method to colposcopy for high-risk (hr) HPV-positive women. **Methods:** We analyzed data from GRECOSELF, a nationwide observational cross-sectional study on HPV primary cervical cancer screening in Greece. Self-collected vaginal samples were tested with the cobas^®^ HPV test (Roche^®^ Molecular Systems, Pleasanton, CA, USA). The Ct value, i.e., the number of cycles needed until DNA amplification occurs exponentially in a PCR, reflects the viral load, and it was evaluated as a triage method to colposcopy for hrHPV-positive women. **Results:** For CIN2 and more advanced lesions, the Ct value, as a dichotomous variable at a cut-off of 29.7, had 54.8% (95%CI: 38.7–70.2) sensitivity, 35.4% (23.9–48.2) Positive Predictive Value (PPV), 74.2% (66.8–80.8) specificity, and 86.4% (73.6–91.6) Negative Predictive Value (NPV) for HPV16/18, while for other hrHPV types, sensitivity was 26.7% (12.3–45.9), PPV 6.7% (2.9–12.8), specificity 78.8% (75.1–82.2), and NPV 95.0% (92.5–96.8). For CIN3 and more advanced lesions, the NPV for non-HPV16/18 was 97.9 (96.1–99.1). **Conclusions:** For self-collected vaginal samples of hrHPV-positive women, the Ct value may be used as a triage method to colposcopy. As Ct values inversely reflect the viral loads, they are lower in high-grade CIN and/or carcinoma.

## 1. Introduction

Cancer of the uterine cervix is one of the three most common infection-related cancers worldwide, together with stomach and liver cancers [1]. Human Papillomavirus (HPV) is a necessary cause of invasive cervical cancer [2]. Persistent infection with high-risk HPV types, most commonly with HPV16 and HPV18, may lead to cervical intraepithelial neoplasia (CIN) and ultimately to invasive cervical cancer. According to world-wide cancer estimates for the year 2022, cervical cancer is the fourth most common cancer in terms of both incidence (*n* = 661,021, 6.8% of all female cancers) and mortality (*n* = 348,189, 8.1% of all female cancer deaths) [1].

Recently, the World Health Organization (WHO) has launched a strategy to eliminate cervical cancer with the goal of reducing its incidence to less than 4 new cases per 100,000 women annually [3]. This strategy includes three interventions: HPV vaccination, screening, and treatment for cervical (pre)cancer [3]. HPV-based screening appears to be more effective than screening with cytology, also known as Papanicolaou’s or Pap test [4], and it is widely used today either in combination with cytology or as a standalone method [5,6,7]. However, many women either hesitate to participate or do not have easy access to HPV screening with samples collected by physicians, and this has led to the development of self-sampling in an effort to overcome these barriers to HPV-based screening [7,8,9]. Obviously, the accuracy of hrHPV assays based on polymerase chain reaction (PCR) in vaginal self-collected specimens may be adversely affected by biological factors such as the viral load [10] and pre-analytical factors such as the sampling, handling, storage, and transportation of specimens; however, evidence suggests that the accuracy of hrHPV assays in self-collected samples is comparable to that of physician-collected specimens [11]. The Ct value, i.e., the number of cycles needed until DNA amplification occurs exponentially in a PCR, reflects the viral load in a given specimen. Previous studies have shown that the Ct value of self-collected samples may be used as a triage method to colposcopy in hrHPV-positive women [12,13], but evidence remains scarce especially in European populations.

In this context, we have conducted the GRECOSELF study investigating HPV DNA testing in self-collected vaginal samples from more than 13,000 women residing in Greece [7,8,9]. The aim of the present secondary analysis was to evaluate the possible use of the HPV viral load, as reflected by the polymerase chain reaction (PCR) cycle threshold (Ct) value, as a triage method to colposcopy for women tested positive for high-risk HPV after self-sampling in the context of the GRECOSELF study.

## 2. Materials and Methods

### 2.1. Data Collection

We analyzed data from GRECOSELF, a nationwide observational cross-sectional study on HPV primary cervical cancer screening, in Greece, on self-collected vaginal samples, using dry cotton swabs [7,8,9], tested with the cobas^®^ HPV test (Roche^®^ Molecular Systems, Pleasanton, CA, USA). In brief, a total of 13,111 non-pregnant women between 25 and 60 years old with an intact uterus and no history of pelvic radiation were enrolled. Data were collected from 12,787 women, recruited between May 2016 and November 2018, from whom a self-collected sample was received by the Institute of Applied Biosciences–Centre for Research and Technology Hellas (INAB/CERTH) giving a valid HPV-DNA result. The study protocol was approved by the Ethics Committees of the Aristotle University of Thessaloniki (237/11.04.2016) and the Centre for Research and Technology Hellas (ETH. COM-9/09-11-2015), and all procedures performed involving human participants were in accordance with the 1964 Helsinki declaration and its later amendments.

According to the GRECOSELF study design [8], hrHPV-positive women were referred for colposcopy to one of the participating academic or national health system hospitals; prior to colposcopy, a cervical sample was taken for cytology. In the case of abnormal colposcopic impression, cervical biopsies were also taken [8]. After colposcopy without cervical biopsy, in the case of no abnormal colposcopic findings, or after the cervical biopsy result was known, women were classified as having cervical disease or not, and treated accordingly. All records included information on age group, laboratory region, and hrHPV status (negative or positive). Histopathological results were retrieved from participating academic or national health system hospitals.

### 2.2. HPV DNA Testing

Self-collected samples were prepared for the Cobas^®^ 4800 system (Roche^®^ Molecular Systems, Pleasanton, CA, USA) according to the manufacturer’s instructions. This fully automated cobas^®^ HPV test is designed for the identification of the 14 hrHPV types (16, 18, 31, 33, 35, 39, 45, 51, 52, 56, 58, 59, 66, and 68) in three channels: HPV16, HPV18, and 12 other types of hrHPV (other hrHPV). The used PCR master mix contains primers for the detection of human beta-globin, which acts as a measure of human cellularity in the specimen. Output from the assay includes a cycle threshold (Ct) value from each of these three channels, corresponding to the number of cycles needed until DNA amplification occurs exponentially in a PCR. Therefore, the Ct value is inversely proportional to the amount of target nucleic acid in the sample, i.e., in the present study, the lower the Ct value, the greater the amount of viral DNA.

### 2.3. Cytology and Histology

The methodologies regarding cytology and histology have been described elsewhere [7]. Briefly, cervicovaginal samples, collected by the physician, were directly immersed in the collection fluid (ThinPrep^®^, Hologic, Bedford, MA, USA). Cytology testing was conducted as per usual practice utilizing the 2014 Bethesda system for cytologic terminology [14,15,16]. Expert cytologists were blinded to the genotyping result and the Ct values.

The gold standard was histological evaluation during colposcopy (colposcopically guided biopsies) [8]. In brief, all cases with abnormal colposcopic impression were subjected to colposcopically guided biopsies. In the case of a type III transformation zone (TZIII), endocervical curettage was performed. Histological findings were classified as normal, cervical intraepithelial neoplasia (CIN) grade 1, CIN2, CIN3, adenocarcinoma in situ (AIS), or invasive cancer, according to international criteria. There were no cases with an invalid histology report. All histopathology samples were evaluated by two expert pathologists, who were semi-blinded, as they were aware that women were high-risk HPV-positive with an abnormal colposcopic impression, but unaware of the genotyping result and the Ct values. If histology was normal, women were referred for cytology after 12 months. If histology was CIN1, women were referred to re-examination with colposcopy after 6 months. If histology was CIN2 or worse (CIN2+), women exited this study and were referred for treatment according to the local cervical precancer treatment guidelines; thus, the proportion of cases confirmed by biopsy versus excision was not further evaluated.

### 2.4. Statistical Analysis

To examine the relation between the Ct values and histopathology, the following exploratory and inferential data analyses were conducted. Histopathology groups were divided as follows: CIN1, CIN2 and CIN3; their comparisons included the following: (a) No Neoplasia (NN) group versus CIN1, CIN2+, and CIN3+, (b) NN and CIN1 versus CIN2+ and CIN3+, (c) CIN1 versus CIN2+ and CIN3+, and (d) CIN2 versus CIN3+, considering only the minimum Ct values of HPV16, HPV18, and other hrHPV-positive samples. The distribution of the Ct values in each histopathology group separately or in groups of different histopathological diagnoses was visualized using histograms and boxplots, and the corresponding descriptive measures (including, min, max, median, IQR, mean, SD) were calculated. The Kruskal–Wallis test was used to examine the correlation between the distribution of Ct values and histopathology. In addition, logistic regression was performed in order to predict histopathology by using the Ct values as a risk factor and having age and smoking status as the possible confounders. To limit the potential technical bias, the Ct value of the β-globin gene, used as a reference in the HPV DNA real-time quantitative PCR assays, was also included as a confounder in the model. The confounding effects were tested by applying stepwise variable selection method with the Akaike Information Criterion (AIC) [17]. The performance of the logistic model was evaluated through the Receiver Operating Characteristic (ROC) curve. No formal sample size calculation was performed because all eligible hrHPV-positive cases with valid histology were included. All statistical tests were two-sided, and the significance level was set to 5%. For statistical analyses, R version 4.1.2 was used.

## 3. Results

### 3.1. Study Population

A total of 15 of 13,111 women participating in the GRECOSELF study were excluded due to unavailable HPV self-sampling and/or unavailable colposcopy. Among the remaining 13,096 women, 1074 (8.2%) were HPV-positive while 12,036 were HPV-negative; therefore, the latter were excluded from further analysis [8].

Of the total 1074 hrHPV-positive women, 300 hrHPV-positive women were excluded because of hrHPV-positive results without colposcopy and/or biopsy. The remaining 774 were examined with colposcopy: of these, 603 did not exhibit any histopathological lesions, either colposcopically (*n* = 529) or histologically (*n* = 74) confirmed (No Neoplasia, NN), 86 had CIN1, 28 CIN2, 42 CIN3, 1 adenocarcinoma, and 1 Squamous Cell Carcinoma (SCC). A lack of histological confirmation in cases of normal colposcopic impression (*n* = 529) may introduce bias; however, it is a standard practice not to take random biopsies in the case of normal colposcopic impression. Thirteen hrHPV-positive women examined with colposcopy were excluded from the analysis because of invalid cytology or invalid HPV testing result in self-sampling. A detailed study flowchart summarizing the number of participants at each stage has been already published elsewhere [8].

### 3.2. Ct Values in hrHPV-Positive Samples

**Figure 1:**
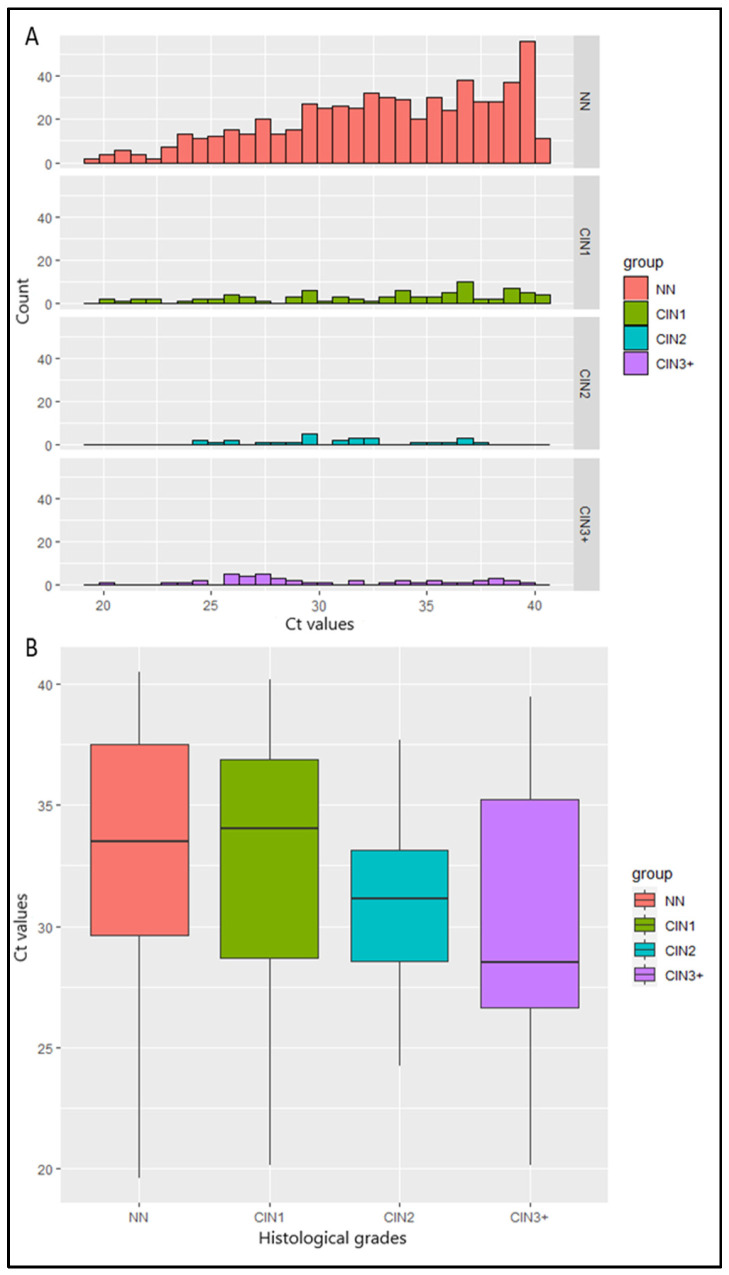
(**A**) Bar plots of Ct values and the number of women with No Neoplasia (NN) and cervical intraepithelial neoplasia of different grades (CIN1, CIN2, CIN3+). (**B**) Boxplots of Ct values and severity of cervical histological grades. Middle line depicts the median. Whisker: min or max. The red box indicates the Ct values of NN, the green box indicates the Ct values of CIN1, the blue box indicates the Ct values of CIN2, and the purple box indicates the Ct values of CIN3+.

Ct values from 774 hrHPV-positive samples were calculated. Median Ct value was lower in women with histology of increasing severity: 33.5 for women with NN, 34 in women with CIN1, 31.2 in women with CIN2, 28.5 in women with CIN3, 31.4 in the woman with adenocarcinoma, and 26.5 in the woman with SCC (*p* = 0.002) (Table 1). Τhe histograms of the Ct values in NN and CIN1 are skewed to the right, in contrast to the corresponding histograms in CIN2 and CIN3+ cases, indicating a relation between the Ct values and the histopathology (Figure 1A). This was also reflected in the boxplots (Figure 1B), in which a reduction in the median Ct value is observed when histopathology’s severity increases. The aforementioned observations were confirmed by the Kruskal–Wallis test (*p* < 0.01). More specifically, post hoc comparisons using Benjamini–Hochberg correction showed that the Ct values in cases with CIN2/CIN3 were significantly lower compared to cases with NN. There was no difference between the Ct values of CIN1 and NN cases. No significant difference was observed in the HPV genotype-specific Ct values: the average Ct values of single HPV16, single HPV18, and other hrHPVs were 33.7, 33.6, and 33.4, respectively (*p* > 0.05) (Table 1).

### 3.3. Ct Value Cut-Off Point Calculation

**Figure 2:**
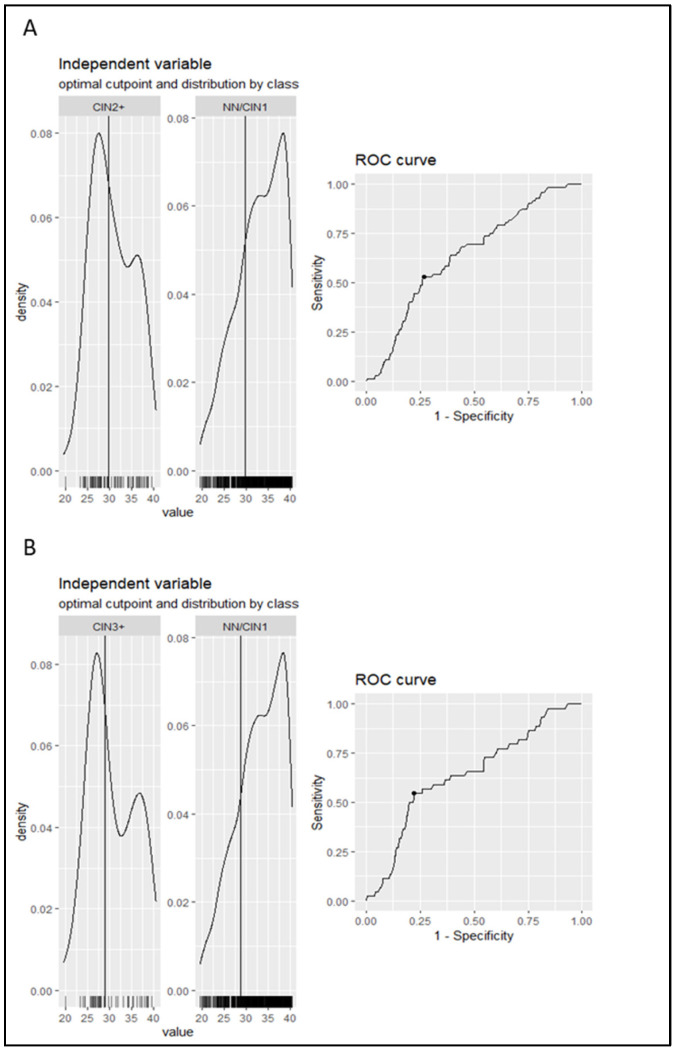
The distribution of the Ct values in each category separately along with the ROC curve obtained from the model are depicted in the graph. The vertical line in the histograms represents the cut-off point. ROC curve and AUC were used to calculate the cut-off of Ct value for cervical intraepithelial neoplasia grade 2 or worse (CIN2+) (**A**) and CIN3+ (**B**) cases versus No Neoplasia (NN)/CIN1. The ROC curve shows the Ct value where sensitivity and specificity are equal to 53% and 73%, respectively, in the case of CIN2+ and 55% and 78%, respectively, in the case of CIN3+. The AUC is equal to 64% (CI: 0.552–0.711) in both comparisons.

The aim of the present analysis was to investigate the performance of Ct value as a triage method to colposcopy of women positive for hrHPV. The first step was to calculate the optimal cut-off point of the Ct value (a continuous variable) and transform it into a dichotomous variable. Therefore, using the ROC curve analysis, the optimal cut-off point, obtained by maximizing either Youden’s index or the product of sensitivity and specificity, was equal to 29.8 for NN/CIN1 vs. CIN2+ and 28.9 for NN/CIN1 vs. CIN3+. The resultant models have a sensitivity of 53% and 55% and a specificity of 73% and 78%, respectively. The area under the curve (AUC) was equal to 0.64 (CI: 0.552–0.711) in both comparisons (Figure 2). The NN and CIN1 groups were unified since no statistically significant difference was observed in the median Ct value between them.

Moreover, a logistic regression model was developed to predict the allocation of a case to the NN group or the CIN2+ group according to Ct values. The model revealed statistical significance (OR = 0.87, *p* < 0.001), which was retained after adjustment for age and smoking status. Because of the decision to group NN cases together with CIN1 cases, we further modified the model to allocate cases to <CIN2 and CIN2+ according to the Ct values. The model revealed statistical significance (OR = 0.92, *p* = 0.006).

### 3.4. Performance of Ct Value for the Triage of HPV16/18-Positive Women to Colposcopy

In the present study, we identified 42 HPV16/18-positive women with CIN2+. Given that, overall, 205 women were positive for HPV16 and/or 18, and these women were included in the analysis, the Positive Predictive Value (PPV) of HPV16/18 genotyping as a triage method to colposcopy for the detection of CIN2+ was 20.5% (95%CI: 15.2–26.7). Cytology identified 17 out of 42 HPV16/18-positive women with CIN2+, i.e., with a sensitivity of 40.5% (95%CI: 25.6–56.7) and a PPV of 70.8% (95%CI: 48.9–87.4). The Ct value, as a dichotomous variable at a cut-off of 29.7, had a sensitivity of 54.8% (95%CI: 38.7–70.2) and a PPV of 35.4% (95%CI: 23.9–48.2), as it identified 23 out of 42 CIN2+ cases with HPV16/18 positivity. An overview of these findings is presented in Table 2.

At the CIN3 threshold, the PPV of HPV16/18 genotyping was 14.1% (95%CI: 9.7–19.7), since among HPV16/18-positive women, we identified 29 with CIN3+. Cytology at the ASCUS threshold had a sensitivity of 37.9% (95%CI: 20.7–57.7) and a PPV of 45.8% (95%CI: 25.6–67.2). The Ct value identified 18 out of 29 cases with CIN3+, with a sensitivity of 62.1% (95%CI: 42.3–79.3) and 27.7% PPV (95%CI: 17.3–40.2). The NPV was above 86% for cytology and Ct value testing, at both thresholds (CIN2 and CIN3). An overview of these findings is presented in Table 2.

### 3.5. Performance of Ct Value for Triage to Colposcopy of hrHPV-Non16/18-Positive Women

In the group of women who were tested positive for hrHPV (non-16/18), we identified 30 women with CIN2+, and 15 of these women had CIN3+. Depending on the disease threshold (CIN2 or CIN3), the PPV of hrHPV-non-16/18 positivity was 5.4% (95%CI: 3.7–7.6) or 2.7% (95%CI: 1.5–4.4), respectively. The Ct value testing detected 8 out of 30 women with CIN2+ (sensitivity: 26.7%, 95%CI 12.3–45.9) and 6 out of 15 women with CIN3+ (sensitivity: 40.0%, 95%CI: 16.3–67.7). The respective values for PPV were 6.7% (95%CI: 2.9–12.8) and 5.0% (95%CI: 1.9–10.7). Cytology had a sensitivity of 40.0% (95%CI: 22.7–59.4) and a PPV of 40.0% (95%CI: 22.7–59.4) at the ASCUS+ cut-off level for the detection of CIN2+ and 20.0% (95%CI: 4.3–48.1) and 10.0% (95%CI: 2.1–2.7) for the detection of CIN3+. An overview of these findings is presented in Table 3.

## 4. Discussion

From a global perspective, cervical cancer is the fourth most common cancer type in women [1], the most common cancer type in 25 countries, and the leading cause of cancer death in 37 countries, mainly in sub-Saharan Africa, South America, and South Eastern Asia [1]. Its incidence and mortality rates are higher in transitioning versus transitioned countries, and this discrepancy is attributed to the higher prevalence of chronic HPV infection and the limited access to screening and vaccination in transitioning countries [1].

Cervical cancer screening is one of the three strategies set by the WHO in order to eliminate cervical cancer [18]. The “screening” goal is to get 70% of the women tested with a high-performance test twice in their lifetime. High-performance tests are HPV nucleic acid-based tests that have been validated as suitable for cervical cancer screening and have been included in screening algorithms along with various triage options [19]. At first, cervical smears for HPV testing were obtained by physicians in the context of clinical examination. HPV self-sampling emerged as a new approach in order to increase cervical cancer screening coverage. It has been demonstrated that the accuracy of hrHPV assays based on polymerase chain reaction (PCR) in vaginal self-collected specimens was comparable to that of specimens collected by physicians [11]. Furthermore, self-sampling was highly accepted by women who are underscreened [9,20,21,22]. Therefore, self-sampling-based strategies are a promising tool in order to achieve wider screening coverage, especially in underserved populations.

Further diagnostic and management protocols in cases of hrHPV detection in physician-collected cervical smears vary depending on whether cytology was performed in the same specimen, as well as on the availability and access to specialist care with colposcopy [23,24]. Likewise, the main question that arises after a woman is tested positive for hrHPV with self-sampling is how she should be managed in terms of either follow-up or further referral. In this context, we have previously compared 17 different strategies combining HPV16/18 genotyping and cytology for the triage to colposcopy of women tested positive for high-risk HPV on self-collected vaginal samples in the GRECOSELF study, a large cross-sectional study with more than 13,000 women participating in Greece [7]. This analysis showed that the optimal strategy was to refer all HPV16-positive women to colposcopy, while women with other hrHPVs only after ASCUS (Atypical Squamous Cells of Unknown Significance) or worse cytology [7].

In the present study, we have conducted a secondary analysis of data from the GRECOSELF study, in order to evaluate the use of the PCR cycle threshold (Ct) value, which reflects the viral load, as a triage method to colposcopy; the Ct values are not proposed as a standalone triage test for HPV-positive women to colposcopy in every setting; they rather represent an additional parameter that could be taken into consideration when further triage is not available or not required (e.g., in HPV16/18-positive women). It is worth noting that the GRECOSELF study was carried out using a comprehensive protocol for the sampling, handling, storage, and transportation of samples, in order to minimize the possible effect of these pre-analytical variables on Ct values. This protocol has been described in detail elsewhere [8]. We have considered two groups of women: those positive for HPV16/18 and those positive for other hrHPVs. Our findings showed that the Ct values presented slightly better sensitivity (and lower PPV) as a triage method to colposcopy, compared to cytology, for HPV16/18-positive women for the detection of CIN2+ or even CIN3+. This would be important in the case of screening algorithms requiring further triage for HPV16/18-positive women. Regarding hrHPV (non-16/18)-positive women, the Ct values performed better than cytology only for the detection of CIN3+ and not for CIN2+. This is important since further triage is always the best option for hrHPV-positive women in any screening setting.

This is, to the best of our knowledge, the first study in Greece and one of the few in a European population evaluating the possible use of the HPV viral load as reflected by the Ct value of self-collected samples as a triage method to colposcopy. Our findings are in line with those of previous studies showing that the Ct value of self-collected samples may be used as a triage method to colposcopy in hrHPV-positive women. In detail, a study analyzed data from 10,498 women participating in a Chinese multi-site screening trial on self-collected samples using the cobas^®^ 4800 HPV assay and found that the Ct values with a cut-off of 33.7, combined with HPV16/18 genotyping, represent a promising triage of HPV-positive women particularly for self-collected samples [12]. Likewise, a large study in the Netherlands analyzed data from 30,808 self-collected and 456,207 clinician-collected specimens and found that the clinical accuracy of hrHPV testing for self-collected samples for the detection of CIN3+ is high [13].

Furthermore, we showed that the viral load increases, which translates into lower Ct values, with an increase in the severity of cervical intraepithelial neoplasia or cervical carcinoma. This is anticipated, and it has also been shown in other studies [13].

The main strength of the present study is that we have analyzed data from a large number of women, i.e., more than 13,000 participants and more than 1000 hrHPV-positive women [7]. Though this study was funded by Roche Diagnostics Hellas who has provided kits and consumables and covered sample transportation costs, the sponsor had no role in data interpretation, analysis, or manuscript preparation. The main limitations as previously noted [7] were that cytology was not available for all women who underwent colposcopy, CIN2 cases were not further triaged with P16 immunostaining, random biopsy or endocervical curettage was not performed in women positive for HPV16 and/or HPV18 with satisfactory colposcopy [7], and, by definition, self-collected samples may vary greatly among individuals in terms of purity, viral load, and the amount of collected material.

In conclusion, in women tested positive for hrHPV types by using the cobas^®^ HPV test on self-collected vaginal samples, the PCR Ct value may be used as a triage method to colposcopy, for example, in settings where cytology is not available. Our findings are in line with previous evidence supporting the view that women tested positive for HPV16/18 should be referred to colposcopy. Larger studies are required to validate if the Ct value could be used as a sole triage test. Nevertheless, the current study sets the basis for using this adjunct tool for risk stratification, particularly within settings where validated triage tests are not readily available or if there is a high risk of loss to follow-up. It should be taken under consideration that, in our study, we investigated the Ct values of self-collected material, a process with great prospects in low- and middle-income settings or for underscreened and unscreened populations. The Ct values, which inversely reflect the viral load, are lower in higher grades of cervical intraepithelial neoplasia or carcinoma.

## Figures and Tables

**Table 1:** Descriptives and Kruskal–Wallis test for Ct values between different categories of histological and HPV test results.

Histology	Number of Women	Median Ct Value	IQR	*p*-Value
NN	603	33.5	29.6–37.5	0.002
CIN1	86	34	28.7–36.9	
CIN2	28	31.2	28.6–33.2	
CIN3	42	28.5	26.8–35.4	
AdenoCa	1	31.4	31.4	
SCC	1	26.5	26.5	
Total	761	-	-	
**HPV Type**	**Number of Women**	**Median Ct Value**	**IQR**	***p*** **-Value**
HPV16	220	33.7	28.4–37.3	>0.05
HPV18	78	33.6	28.8–37	
hrHPV-non-16/18	874	33.4	29.4–37.1	
Total	1172			

NN: No Neoplasia; CIN: cervical intraepithelial neoplasia; AdenoCa: adenocarcinoma; SCC: Squamous Cell Carcinoma; IQR: interquartile range; hrHPV: high-risk Human Papillomavirus.

**Table 2:** Performance of Ct values of the cobas^®^ HPV test as a triage test to colposcopy for HPV16- and/or HPV18-positive women.

	Sensitivity *n*/*N*, (%) 95%CI	Specificity *n*/*N*, (%) 95%CI	PPV *n*/*N*, (%) 95%CI	NPV *n*/*N*, (%) 95%CI
**CIN2+ Threshold**				
**HPV16/18**	(42/42) 100.0 (100.0–100.0)	-	(42/205) 20.5 (15.2–26.7)	-
ASCUS+ *	(17/42) 40.5 (25.6–56.7)	(156/163) 95.7 (91.4–98.3)	(17/24) 70.8 (48.9–87.4)	(156/181) 86.2 (80.3–90.9)
**Ct value** **(Cut-off: 29.7)**	(23/42) 54.8 (38.7–70.2)	(121/163) 74.2 (66.8–80.8)	(23/65) 35.4 (23.9–48.2)	(121/140) 86.4 (73.6–91.6)
**CIN3+ Threshold**				
**HPV16/18**	(29/29) 100.0 (100.0–100.0)	-	(29/205) 14.1 (9.7–19.7)	-
**ASCUS+**	(11/29) 37.9 (20.7–57.7)	(163/176) 92.6 (87.7–96.0)	(11/24) 45.8 (25.6–67.2)	(163/181) 90.1 (84.7–94.0)
**Ct value** **(Cut-off: 29.7)**	(18/29) 62.1 (42.3–79.3)	(129/176) 73.3 (66.1–79.7)	(18/65) 27.7 (17.3–40.2)	(129/140) 92.1 (86.4–96.0)

CIN: cervical intraepithelial neoplasia; PPV: Positive Predictive Value; NPV: Negative Predictive Value; HPV: Human Papillomavirus; ASCUS: Atypical Squamous Cells of Undetermined Significance. * Pap test was not available for all women subjected to colposcopy. Calculations were based on the available data.

**Table 3:** Performance of Ct values of the cobas HPV test as a triage test for high-risk HPV other than HPV16- and/or HPV18-positive women to colposcopy.

	Sensitivity *n*/*N*, (%) 95%CI	Specificity *n*/*N*, (%) 95%CI	PPV *n*/*N*, (%) 95%CI	NPV *n*/*N*, (%) 95%CI
**CIN2+ Threshold**				
**hrHPV-non-16/18**	(30/30) 100.0 (100.0–100.0)	-	(30/558) 5.4 (3.7–7.6)	-
ASCUS+ *	(12/30) 40.0 (22.7–59.4)	(510/528) 96.6 (94.7–98.0)	(12/30) 40.0 (22.7–59.4)	(510/528) 96.6 (94.7–98.0)
**Ct value** **(Cut-off: 29.7)**	(8/30) 26.7 (12.3–45.9)	(417/529) 78.8 (75.1–82.2)	(8/119) 6.7 (2.9–12.8)	(417/439) 95.0 (92.5–96.8)
**CIN3+ Threshold**				
**hrHPV-non-16/18**	(15/15) 100.0 (100.0–100.0)	-	(15/558) 2.7 (1.5–4.4)	-
**ASCUS+**	(3/15) 20.0 (4.3–48.1)	(516/543) 95.0 (92.8–96.7)	(3/30) 10.0 (2.1–2.7)	(516/528) 97.7 (96–98.8)
**Ct value** **(Cut-off: 29.7)**	(6/15) 40.0 (16.3–67.7)	(430/543) 79.2 (75.5–82.5)	(6/119) 5.0 (1.9–10.7)	(430/439) 97.9 (96.1–99.1)

CIN: cervical intraepithelial neoplasia; PPV: Positive Predictive Value; NPV: Negative Predictive Value; HPV: Human Papillomavirus; ASCUS: Atypical Squamous Cells of Undetermined Significance. * Pap test was not available for all women subjected to colposcopy. Calculations were based on the available data.

## Data Availability

The original contributions presented in this study are included in the article. Further inquiries can be directed to the corresponding authors.
